# Supplementary material for: SARS-CoV and SARS-CoV-2 are transmitted through the air between ferrets over more than one meter distance
Source: Nat Commun. 2021 Mar 12;12:1653. doi: 10.1038/s41467-021-21918-6 (PMC7955093; doi:10.1038/s41467-021-21918-6)
Supplement: Supplementary file 1 — Supplementary Information [file 41467_2021_21918_MOESM1_ESM.pdf]

Supplementary information for

SARS-CoV and SARS-CoV-2 are transmitted through the air between ferrets over more than one meter distance

Jasmin S Kutter[1], Dennis de Meulder[1], Theo M Bestebroer[1], Pascal Lexmond[1], Ard Mulders[1], Mathilde Richard[1], Ron AM Fouchier[1], Sander Herfst[1]

[1]Department of Viroscience, Erasmus University Medical Center, Rotterdam, The Netherlands.

Table S1. Detection of single nucleotide polymorphisms by Illumina sequencing

a. Single nucleotide polymorphisms in the ferret samples present at >5%

	Time after inoculation (dpi)/exposure (dpe)	Nt change	Aa change	Protein	Reads with the specified substitution (%)
Donor ferret 1	3 dpi	A23032C<br>A23586G	N501T<br>S686G	Spike<br>Spike	95.0<br>96.8
Donor ferret 2	3 dpi	G228T<br>A23032C<br>A23586G	-<br>N501T<br>S686G	NA (5'UTR)<br>Spike<br>Spike	8.3<br>53.0<br>84.3
Donor ferret 3	3 dpi	A23032C<br>A23586G<br>T29552A	N501T<br>S686G<br>F9L	Spike<br>Spike<br>ORF10	98.0<br>98.3<br>15.2
Donor ferret 4	3 dpi	G228T<br>C5790T<br>A23032C<br>A23586G	-<br>L1035F<br>N501T<br>S686G	NA (5'UTR)<br>Nsp3<br>Spike<br>Spike	6.1<br>34.0<br>98.8<br>99.0
Indirect recipient ferret 3	7 dpe	A23032C<br>A23586G<br>G29719T	N501T<br>S686G<br>-	Spike<br>Spike<br>NA (3'UTR)	99.6<br>99.6<br>5.8
Indirect recipient ferret 4	5 dpe	G228T<br>C10757T<br>A23032C<br>A23586G	-<br>D245D<br>N501T<br>S686G	NA (5'UTR)<br>Nsp5<br>Spike<br>Spike	5.3<br>99.3<br>99.8<br>99.2

b. Presence of the ferret sample single nucleotide polymorphisms in the virus stocks (>1%)

			Reads with the specified substitution (%)	
Nt change	Aa position	Protein	Virus stock P1	Virus stock P3 VeroE6
G228T	-	5'UTR	<1	<1
C5790T	L1035F	Nsp3	<1	<1
C10757T	D245D	Nsp5	<1	<1
A23032C	N501T	Spike	2.0	<1
A23586G	S686G	Spike	15.2	8.1
T29552A	F9L	ORF10	<1	<1
G29719T	-	3'UTR	<1	<1

Nt: nucleotide; Aa: amino acid; NA: not applicable

Table S2. Sequences of primers and probes used for qRT-PCR analysis

Virus	Oligo name & orientation	Sequence (5'-3')
A/H1N1 virus	Forward primer	CTTCTRACCGAGGTCGAAACGTA
	Reverse primer	TCTTGTCTTTAGCCAYTCCATGAG
	Probe	FAM-TCAGGCCCCCTCAAAGCCGAGA-BHQ
SARS-CoV-2	Forward primer	ACAGGTACGTTAATAGTTAATAGCGT
	Reverse primer	ATATTGCAGCAGTACGCACACA
	Probe	FAM-ACACTAGCCATCCTTACTGCGCTTCG-BHQ
SARS-CoV	Forward primer	CAAACATTGGCCG CAAATT
	Reverse primer	CAATGCGTGACA TTCCAAAGA
	Probe	FAM-CACAATTTGCTCCAAGTGCCTCTGCA-BHQ
PDV	Forward primer	CGGGTGCCTTTTACAAGAAC
	Reverse primer	TTCTTTCCTCAACCTCGTCC
	Probe	Cy5-ATGCAAGGGCCAATTCTTCCAAGTT-BHQ

Table S3. Sequences of primers used for the generation of amplicons for wholegenome sequencing and next-generation sequencing

Mix 1

Name	Sequence	Orientation	Volume
SARS-CoV-2_1_LEFT	ACCAACCAACTTTCGATCTCTTGT	FWD	10µl
SARS-CoV-2_1_RIGHT	CGAGCATCCGAACGTTTGATGA	REV	10µl
SARS-CoV-2_3_LEFT	ACGAGCTTGGCACTGATCCTTA	FWD	10µl
SARS-CoV-2_3_RIGHT	GGTTGCATTCATTTGGTGACGC	REV	10µl
SARS-CoV-2_5_LEFT	TGTCCAGCATGTCACAATTCAGA	FWD	10µl
SARS-CoV-2_5_RIGHT	TACAACACGAGCAGCCTCTGAT	REV	10µl
SARS-CoV-2_7_LEFT	TGGCACTGTTTATGAAAAACTCAAACC	FWD	10µl
SARS-CoV-2_7_RIGHT	TTTCGAGCAACATAAGCCCGTT	REV	10µl
SARS-CoV-2_9_LEFT	TCACTTTTGAACTTGATGAAAGGATTGA	FWD	10µl
SARS-CoV-2_9_RIGHT	GATTGTCCTCACTGCCGTCTTG	REV	10µl
SARS-CoV-2_11_LEFT	AAACATGGAGGAGGTGTTGCAG	FWD	20µl
SARS-CoV-2_11_RIGHT	TCTTGTTTTCTCTGTTCAACTGAAGGT	REV	20µl
SARS-CoV-2_13_LEFT	GCTCCATATATAGTGGGTGATGTTGT	FWD	10µl
SARS-CoV-2_13_RIGHT	AGCCATGTGTTACATAGCCAAGTG	REV	10µl
SARS-CoV-2_15_LEFT	CTATTCTGGACAATCTACACAACTAGGT	FWD	10µl
SARS-CoV-2_15_RIGHT	AGCATCTTGTAGAGCAGGTGGA	REV	10µl
SARS-CoV-2_17_LEFT	GCTGTTATGTACATGGGCACACT	FWD	10µl
SARS-CoV-2_17_RIGHT	GCTTGCGTTTGGATATGGTTGG	REV	10µl
SARS-CoV-2_19_LEFT	TGTTACATAAACCTATTGTTTGGCATGT	FWD	10µl
SARS-CoV-2_19_RIGHT	TCCCAAGGGACACTATTAACAGCA	REV	10µl
SARS-CoV-2_21_LEFT	AGCAAAGAATACTGTTAAGAGTGTCGG	FWD	20µl
SARS-CoV-2_21_RIGHT	TCGGGGCCATTTGTACAAGATT	REV	20µl
SARS-CoV-2_23_LEFT	ACTATTGTTAATGGTGTTAGAAGGTCCT	FWD	10µl
SARS-CoV-2_23_RIGHT	GCAACTTCCGCACTATCACCAA	REV	10µl
SARS-CoV-2_25_LEFT	TGTCTTAAATTGTCACATCAATCTGACAT	FWD	10µl
SARS-CoV-2_25_RIGHT	TCACGAGTGACACCACCATCAA	REV	10µl
SARS-CoV-2_27_LEFT	CTGGTTTGCCTGGCACGATATT	FWD	10µl
SARS-CoV-2_27_RIGHT	TCTACACCACAGAAAACTCCTGGT	REV	10µl
SARS-CoV-2_29_LEFT	ACAGTCATGTAGTTGCCTTTAATACTTTAC	FWD	10µl
SARS-CoV-2_29_RIGHT	GAGCCTTTGCGAGATGACAACA	REV	10µl
SARS-CoV-2_31_LEFT	AACGGTCTTTGGCTTGATGACG	FWD	10µl
SARS-CoV-2_31_RIGHT	ACCTTCTAAGTCTGTGCCAGCA	REV	10µl
SARS-CoV-2_33_LEFT	TGTGGCTATGAAGTACAATTATGAACCT	FWD	10µl
SARS-CoV-2_33_RIGHT	AGCTACAGTGGCAAGAGAAGGT	REV	10µl
SARS-CoV-2_35_LEFT	TGGTGCTAGGAGAGTGTGGACA	FWD	10µl
SARS-CoV-2_35_RIGHT	GGCTACTTTGATACAAGGTTTGCC	REV	10µl
SARS-CoV-2_37_LEFT	CTTTCCATGCAGGGTGCTGTAG	FWD	10µl
SARS-CoV-2_37_RIGHT	GTTTGGCTGCTGTTGTAAGAGGT	REV	10µl
SARS-CoV-2_39_LEFT	ACAATTCACCTAATTTAGCATGGCCT	FWD	10µl
SARS-CoV-2_39_RIGHT	TTGGTTGTCCCCCACTAGCTAG	REV	10µl
SARS-CoV-2_41_LEFT	AGTATGTACAAATACCTACAACTTGTGCT	FWD	10µl
SARS-CoV-2_41_RIGHT	AGCATAGACGAGGTCTGCCATT	REV	10µl
SARS-CoV-2_43_LEFT	TGGTATTGTTGGTGTACTGACATTAGA	FWD	10µl
SARS-CoV-2_43_RIGHT	AGGGTCAGCAGCATACACAAGT	REV	10µl
SARS-CoV-2_45_LEFT	CACTTCTTCTTTGCTCAGGATGGT	FWD	10µl
SARS-CoV-2_45_RIGHT	AACATGTTGTGCCAACCACCAT	REV	10µl
SARS-CoV-2_47_LEFT	TGTAGCTTGTCACACCGTTTCT	FWD	10µl
SARS-CoV-2_47_RIGHT	AGTAAGGTCAGTCTCAGTCCAACA	REV	10µl
SARS-CoV-2_49_LEFT	AGGAGTATGCTGATGTCTTTCATTTGT	FWD	10µl
SARS-CoV-2_49_RIGHT	CACCAGCATTTGTCCAGTCACA	REV	10µl
SARS-CoV-2_51_LEFT	TCTTTCATGGGAAGTTGGTAAACCT	FWD	20µl
SARS-CoV-2_51_RIGHT	TCACATAGTGCATCAACAGCGG	REV	20µl
SARS-CoV-2_53_LEFT	TGTCAATGCCAGATTAYGTGCT	FWD	10µl
SARS-CoV-2_53_RIGHT	CAAGAGTGAGCTGTTTCAGTGGT	REV	10µl

SARS-CoV-2_55_LEFT	TGTTGACACTAAATTCAAAACTGAAGGT	FWD	20µl
SARS-CoV-2_55_RIGHT	TGTCAACTCAAAGCCATGTGCC	REV	20µl
SARS-CoV-2_57_LEFT	CGTTTATGATTGATGTTCAACAATGGGG	FWD	10µl
SARS-CoV-2_57_RIGHT	ACAACCAGGCAAGTTAAGGTTAGA	REV	10µl
SARS-CoV-2_59_LEFT	AGTCTCATGGAAAACAAGTAGTGTCA	FWD	10µl
SARS-CoV-2_59_RIGHT	ATTAGCAGCAATGTCCACACCC	REV	10µl
SARS-CoV-2_61_LEFT	AGAAATGCCCGTAATGGTGTTCT	FWD	10µl
SARS-CoV-2_61_RIGHT	TGAACCTGTTTGCGCATCTGTT	REV	10µl
SARS-CoV-2_63_LEFT	TGGCCATGTAGAAACATTTTACCCA	FWD	10µl
SARS-CoV-2_63_RIGHT	ATAGCCACGGAACCTCCAAGAG	REV	10µl
SARS-CoV-2_65_LEFT	GGCAAACCACGCGAACAAATAR	FWD	10µl
SARS-CoV-2_65_RIGHT	ACCTCTTAGTACCATTGGTCCCA	REV	10µl
SARS-CoV-2_67_LEFT	CAATTTTGTAATGATCCATTTTTGGGTGT	FWD	10µl
SARS-CoV-2_67_RIGHT	GGTCAAGTGCACAGTCTACAGC	REV	10µl
SARS-CoV-2_69_LEFT	ACTGTGTTGCTGATTATTCTGTCCT	FWD	20µl
SARS-CoV-2_69_RIGHT	TAGGTCCACAAACAGTTGCTGG	REV	20µl
SARS-CoV-2_71_LEFT	ACTTCTAACCAGGTTGCTGTTCTT	FWD	10µl
SARS-CoV-2_71_RIGHT	CAGCTATTCCAGTTAAAGCACGGT	REV	10µl
SARS-CoV-2_73_LEFT	CTTGCAGATGCTGGCTTCATCA	FWD	10µl
SARS-CoV-2_73_RIGHT	TGCACTTCAGCCTCAACTTTGT	REV	10µl
SARS-CoV-2_75_LEFT	CTTCCCTCAGTCAGCACCTCAT	FWD	10µl
SARS-CoV-2_75_RIGHT	CAAGCCAGCTATAAAACCTAGCCA	REV	10µl
SARS-CoV-2_77_LEFT	TGGAACTGTAACTTTGAAGCAAGGT	FWD	10µl
SARS-CoV-2_77_RIGHT	GACTTGTTGTGCCATCACCTGA	REV	10µl
SARS-CoV-2_79_LEFT	GGTGTTGAACATGTTACCTTCTTCAT	FWD	10µl
SARS-CoV-2_79_RIGHT	GTACCGTTGGAATCTGCCATGG	REV	10µl
SARS-CoV-2_81_LEFT	CTTGTTTTGTGCTTGCTGCTGT	FWD	10µl
SARS-CoV-2_81_RIGHT	ACTGCTACTGGAATGGTCTGTGT	REV	10µl
SARS-CoV-2_83_LEFT	TGAAGAGCAACCAATGGAGATTGA	FWD	10µl
SARS-CoV-2_83_RIGHT	TGTTCGTTTAGGCGTGACAAGT	REV	10µl
SARS-CoV-2_85_LEFT	AGCACCTTTAATTGAATTGTGCGTG	FWD	10µl
SARS-CoV-2_85_RIGHT	CGTCTGGTAGCTCTTCGGTAGT	REV	10µl
SARS-CoV-2_87_LEFT	AAAAGATCACATTGGCACCCGC	FWD	10µl
SARS-CoV-2_87_RIGHT	CGACATTCCGAAGAACGCTGAA	REV	10µl
SARS-CoV-2_89_LEFT	AGGCTGATGAAACTCAAGCCTT	FWD	20µl
SARS-CoV-2_89_RIGHT	AAAATCACATGGGGATAGCACTACT	REV	20µl

Mix	2		
Name	Sequence	Orientation	Volume
SARS-CoV-2_2_LEFT	TCGTACGTGGCTTTGGAGACTC	FWD	10µl
SARS-CoV-2_2_RIGHT	ATGCACTCAAGAGGGTAGCCAT	REV	10µl
SARS-CoV-2_4_LEFT	ACACCTTCAATGGGGAATGTCC	FWD	10µl
SARS-CoV-2_4_RIGHT	AGGCACACTTGTTATGGCAACC	REV	10µl
SARS-CoV-2_6_LEFT	TGTGAAAGGTTTGGATTATAAAGCATTCA	FWD	10µl
SARS-CoV-2_6_RIGHT	ACAGGTGACAATTTGTCCACCG	REV	10µl
SARS-CoV-2_8_LEFT	AGGGAGAAACACTTCCCACAGA	FWD	10µl
SARS-CoV-2_8_RIGHT	AATCAATGCCCAGTGGTGTAAGT	REV	10µl
SARS-CoV-2_10_LEFT	TGAGTATGGTACTGAAGATGATTACCAAG	FWD	10µl
SARS-CoV-2_10_RIGHT	GCCGACAACATGAAGACAGTGT	REV	10µl
SARS-CoV-2_12_LEFT	ACTGTTCGCACGAAYGTCTACT	FWD	10µl
SARS-CoV-2_12_RIGHT	CCTGACCCGGGTAAGTGGTTAT	REV	10µl
SARS-CoV-2_14_LEFT	GTTTCAACTATACAGCGTAAATATAAGGGT	FWD	20µl
SARS-CoV-2_14_RIGHT	CGTGTGGAGGTTAATGTTGTCTACT	REV	20µl
SARS-CoV-2_16_LEFT	AGGTACATGTCAGCATTAAATCACACT	FWD	10µl
SARS-CoV-2_16_RIGHT	AGTTCATACTGAGCAGGTGGTG	REV	10µl
SARS-CoV-2_18_LEFT	CAGTTACACAACAACCATAAAACCAGT	FWD	10µl
SARS-CoV-2_18_RIGHT	GATTATCCATTCCCTGCGCGTC	REV	10µl
SARS-CoV-2_20_LEFT	TACAGAAGAGGTTGGCCACACA	FWD	7.5µl
SARS-CoV-2_20_RIGHT	AACACYTAAAGCAGCGGTTGAG	REV	7.5µl
SARS-CoV-2_22_LEFT	AGTTGCAGAGTGGTTTTTGGCA	FWD	10µl
SARS-CoV-2_22_RIGHT	ACTGTAGTGACAAGTCTCTCGCA	REV	10µl
SARS-CoV-2_24_LEFT	AGCTAATAACACTAAAGGTTCATTGCCT	FWD	10µl
SARS-CoV-2_24_RIGHT	TGACTTTTTGCTACCTGCGCAT	REV	10µl
SARS-CoV-2_26_LEFT	TGGTTGAAGCAGTTAATTAAAGTTACACT	FWD	10µl
SARS-CoV-2_26_RIGHT	TTCAGCAGCCAAAACACAAGCT	REV	10µl
SARS-CoV-2_28_LEFT	CCTTGAAGGTTCTGTTAGAGTGGT	FWD	10µl
SARS-CoV-2_28_RIGHT	AGGTGTGAACATAACCATCCACTG	REV	10µl
SARS-CoV-2_30_LEFT	AGAAATGTATCTAAAGTTGCGTAGTGATG	FWD	10µl
SARS-CoV-2_30_RIGHT	CCCTGAGTTGAACATTACCAGCC	REV	10µl
SARS-CoV-2_32_LEFT	TACCAATGTGCTATGAGGCCCA	FWD	10µl
SARS-CoV-2_32_RIGHT	GCACTACCCAATATGGTACGTCC	REV	10µl
SARS-CoV-2_34_LEFT	GTCCAGAGTACTCAATGGTCTTTGT	FWD	10µl
SARS-CoV-2_34_RIGHT	ACCTCTGGCCAAAAACATGACA	REV	10µl
SARS-CoV-2_36_LEFT	CGCTACTTTAGACTGACTCTTGGTG	FWD	10µl
SARS-CoV-2_36_RIGHT	ATCACCATTAGCAACAGCCTGC	REV	10µl
SARS-CoV-2_38_LEFT	AGATCTGAGGACAAGAGGGCAA	FWD	10µl
SARS-CoV-2_38_RIGHT	TGTCATCAGTGCAAGCAGTTTGT	REV	10µl
SARS-CoV-2_40_LEFT	GGTATGGTACTTGGTAGTTTAGCTGC	FWD	10µl
SARS-CoV-2_40_RIGHT	ACGATTGTGCATCAGCTGACTG	REV	10µl
SARS-CoV-2_42_LEFT	TCTCTAACTACCAACATGAAGAAACAATTT	FWD	20µl
SARS-CoV-2_42_RIGHT	GCAGTTAAAGCCCTGGTCAAGG	REV	20µl
SARS-CoV-2_44_LEFT	TGGACCACTAGTGAGAAAAATATTTGTTG	FWD	10µl
SARS-CoV-2_44_RIGHT	ACAGCCACCATCGTAACAATCA	REV	10µl
SARS-CoV-2_46_LEFT	TGCAAAGAATAGAGCTCGCACC	FWD	10µl
SARS-CoV-2_46_RIGHT	TGCATTAACATTGGCCGTGACA	REV	10µl
SARS-CoV-2_48_LEFT	CTCTCTGACGATGCTGTTGTGT	FWD	10µl
SARS-CoV-2_48_RIGHT	TGCGGTGTGTACATAGCCTCAT	REV	10µl
SARS-CoV-2_50_LEFT	AGGAGGTATGAGCTATTATTGTAAATCACA	FWD	10µl
SARS-CoV-2_50_RIGHT	GTTGTACCTCGGTAAACAACAGCA	REV	10µl
SARS-CoV-2_52_LEFT	TGCAAATTATCAAAAGGTTGGTATGCA	FWD	10µl
SARS-CoV-2_52_RIGHT	CCGAGGAACATGTCTGGACCTA	REV	10µl
SARS-CoV-2_54_LEFT	TGGAGAAAAGCTGTCTTTATTTCACCT	FWD	10µl
SARS-CoV-2_54_RIGHT	GCTTCTTCGCGGGTGATAAACA	REV	10µl
SARS-CoV-2_56_LEFT	ACCACCGCCTGGAGATCAATTT	FWD	10µl
SARS-CoV-2_56_RIGHT	CGCTTAACAAAGCACTCGTGGA	REV	10µl

SARS-CoV-2_58_LEFT	GCCTTGTAGTGACAAAGCTTATAAAATAGA	FWD	10µl
SARS-CoV-2_58_RIGHT	AAACCCACAAGCTAAAGCCAGC	REV	10µl
SARS-CoV-2_60_LEFT	CAGGGTGAAGTACCAGTTTCTATCATT	FWD	10µl
SARS-CoV-2_60_RIGHT	GAGTAAAGTAAGTTTCAGGTAATTGTTGG	REV	10µl
SARS-CoV-2_62_LEFT	TCGTTTATGGAGATTTTAGTCATAGTCAGT	FWD	10µl
SARS-CoV-2_62_RIGHT	TTGCGACATTCATCATTATGCCTTT	REV	10µl
SARS-CoV-2_64_LEFT	CTGTACATACAGCTAATAAATGGGATCTCA	FWD	10µl
SARS-CoV-2_64_RIGHT	TTTGACCTTCTTTTAAAGACATAACAGCA	REV	10µl
SARS-CoV-2_66_LEFT	ACCCCCTGCATACACTAATTCTYT	FWD	10µl
SARS-CoV-2_66_RIGHT	ACCCTGTTTTCCTTCAAGGTCC	REV	10µl
SARS-CoV-2_68_LEFT	ACATAGAAGTTATTTGACTCCTGGTGA	FWD	10µl
SARS-CoV-2_68_RIGHT	CCCTGGAGCGATTTGTCTGACT	REV	10µl
SARS-CoV-2_70_LEFT	CCGGTAGCACACCTTGTAATGG	FWD	10µl
SARS-CoV-2_70_RIGHT	CCCCTATTAAACAGCCTGCACG	REV	10µl
SARS-CoV-2_72_LEFT	TGTTACCACAGAAATTCTACCAGTGT	FWD	10µl
SARS-CoV-2_72_RIGHT	TACCCGCTAACAGTGCAGAAGT	REV	10µl
SARS-CoV-2_74_LEFT	GTGCACTTGGAAAACTTCAAGATGT	FWD	10µl
SARS-CoV-2_74_RIGHT	TGTTACAAACCAGTGTGTGCCA	REV	10µl
SARS-CoV-2_76_LEFT	GTTGATTTAGGTGACATCTCTGGCA	FWD	7.5µl
SARS-CoV-2_76_RIGHT	AGCGCTCTGAAAAACAGCAAGA	REV	7.5µl
SARS-CoV-2_78_LEFT	CTTTGGCTTTGCTGGAAATGCC	FWD	10µl
SARS-CoV-2_78_RIGHT	GTGCTTACAAAGGCACGCTAGT	REV	10µl
SARS-CoV-2_80_LEFT	ACGTGAGTCTTGTAAAACCTTCTTTTT	FWD	10µl
SARS-CoV-2_80_RIGHT	AATGACCACATGGAACGCGTAC	REV	10µl
SARS-CoV-2_82_LEFT	GGACCTGCCTAAAGAAATCACTGT	FWD	10µl
SARS-CoV-2_82_RIGHT	TGCCCTCGTATGTTCCAGAAGA	REV	10µl
SARS-CoV-2_84_LEFT	CTTCACACTCAAAAGAAAGACAGAATGA	FWD	10µl
SARS-CoV-2_84_RIGHT	ACGAACAACGCACTACAAGACT	REV	10µl
SARS-CoV-2_86_LEFT	GGCCCCAAGGTTTACCCAATAA	FWD	10µl
SARS-CoV-2_86_RIGHT	CTGTTGCGACTACGTGATGAGG	REV	10µl
SARS-CoV-2_88_LEFT	TAACACAAGCTTTCGGCAGACG	FWD	10µl
SARS-CoV-2_88_RIGHT	GTGGTCTGCATGAGTTTAGGCC	REV	10µl

FWD: forward; REV: reverse

a

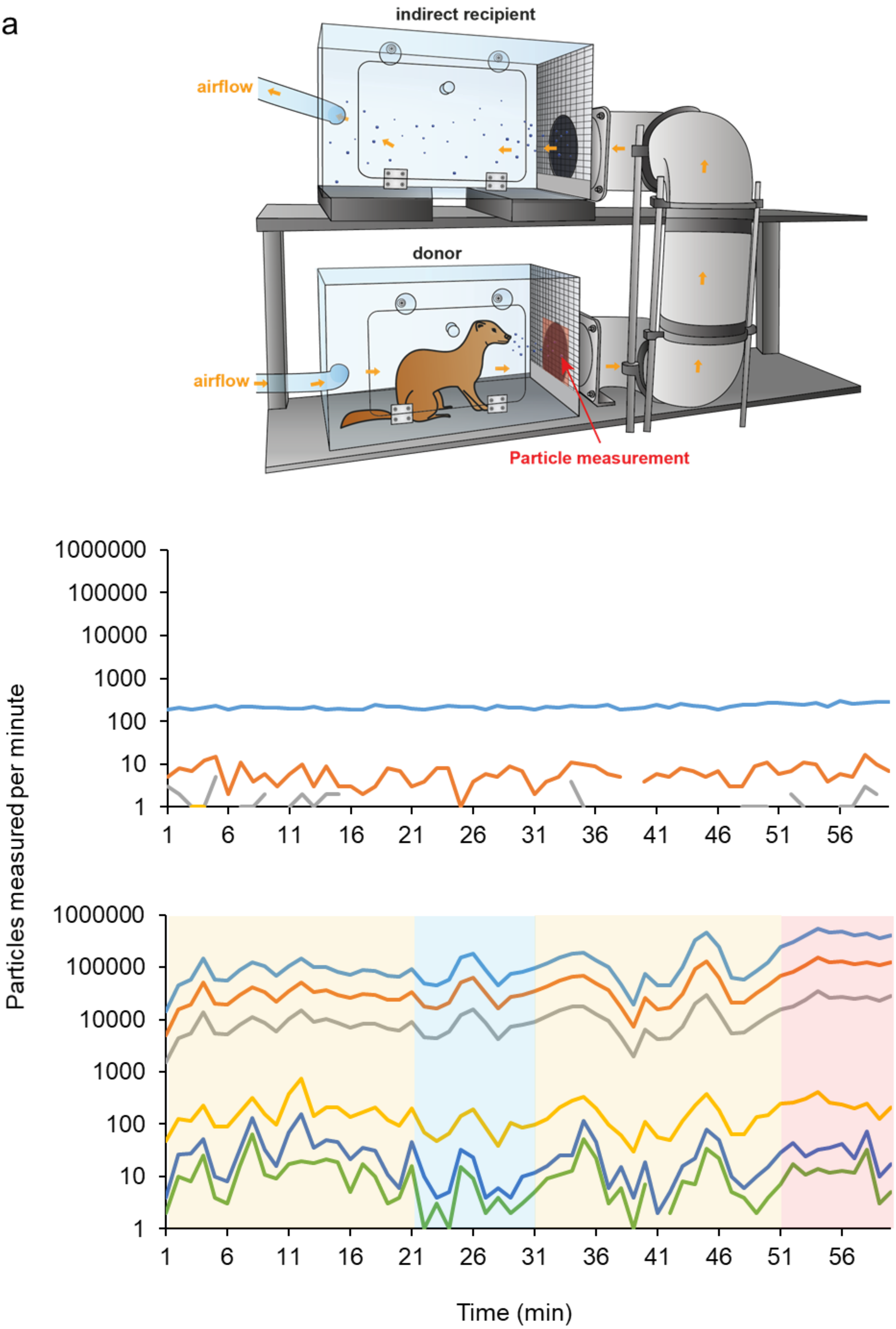
indirect recipient
airflow
donor
airflow
Particle measurement
1000000
100000
10000
1000
100
10
1
1 6 11 16 21 26 31 36 41 46 51 56
Particles measured per minute
Time (min)

b

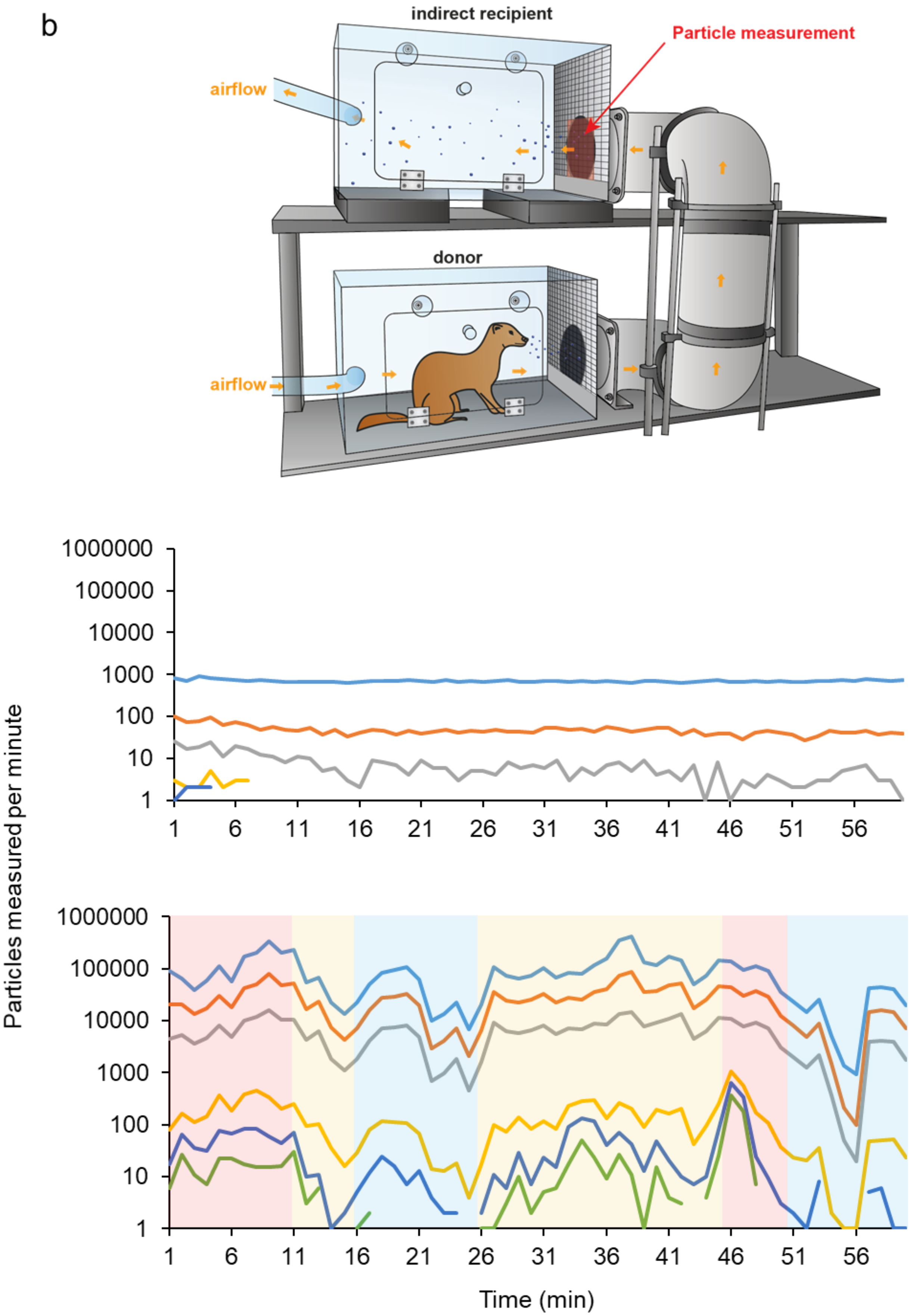
indirect recipient
Particle measurement
airflow
donor
airflow
1000000
100000
10000
1000
100
10
1
1 6 11 16 21 26 31 36 41 46 51 56
Particles measured per minute
1000000
100000
10000
1000
100
10
1
1 6 11 16 21 26 31 36 41 46 51 56
Time (min)

Figure S1. Number and particle size distribution of aerosols and droplets in the experimental set-up. The number and size of particles entering (a) and exiting (b) the tubing system was recorded continuously with an aerodynamic particle sizer (APS) every minute for a total of 60 min. Measurements were performed without (upper graph) and with (bottom graph) one uninfected ferret present in the bottom cage to determine the number and size distribution of particles (0.3 µm: light blue, 0.5 µm: orange, 1.0 µm: grey, 3.0 µm: yellow, 5.0 µm: dark blue and 10.0 µm green) originating from the caging environment itself and from the ferret. In order to correlate the amount and size of particles with the activity of the ferret, the animal's behavior was recorded every 5 minutes. The activity was scored based on three categories and shown as color shading in the bottom graphs: calm (blue), active (yellow) and agitated (red). Calm included resting, occasional grooming; active included moving around, exploring, occasional scratching of walls and carpet; agitated included agitated movement, intense scratching of walls and carpet. Source data are provided as a Source Data file.

a

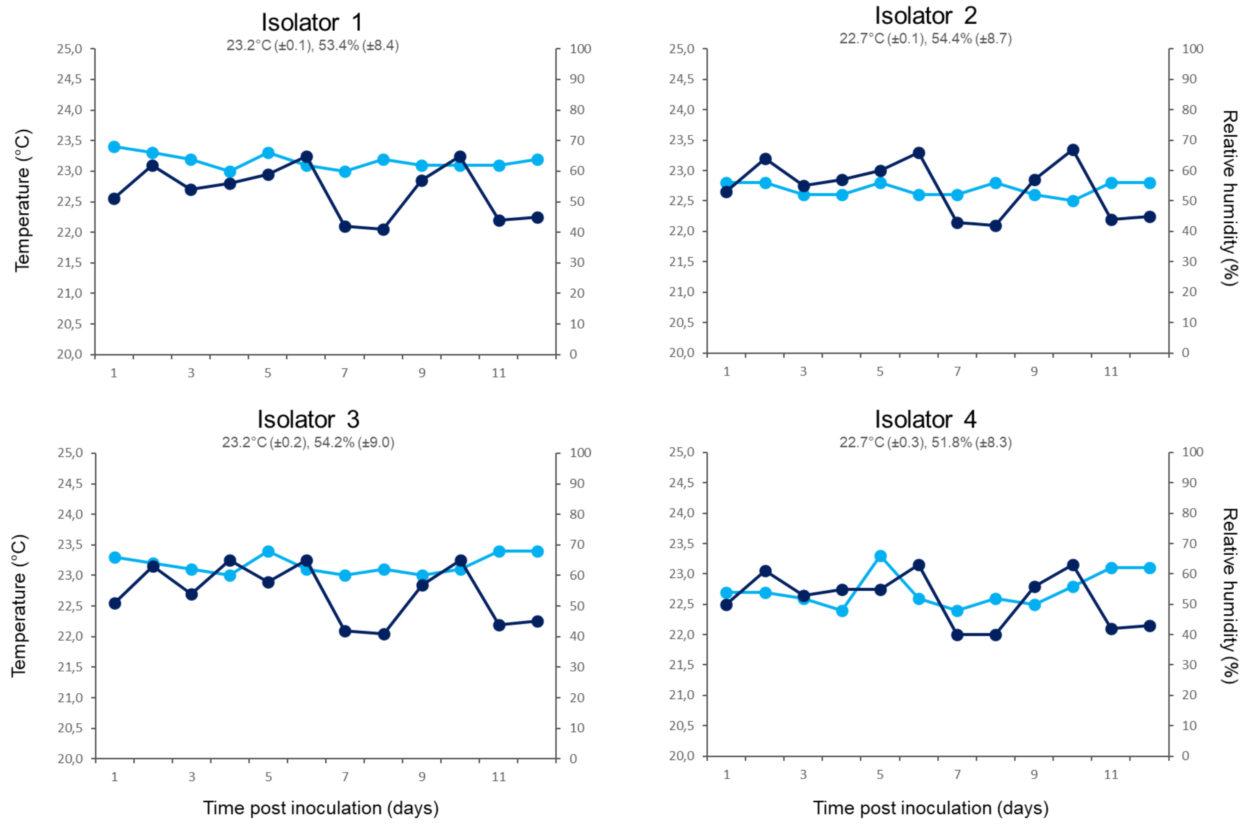
Isolator 1
23.2°C (±0.1), 53.4% (±8.4)
Isolator 2
22.7°C (±0.1), 54.4% (±8.7)
Isolator 3
23.2°C (±0.2), 54.2% (±9.0)
Isolator 4
22.7°C (±0.3), 51.8% (±8.3)
Temperature (°C)
Relative humidity (%)
Time post inoculation (days)

b

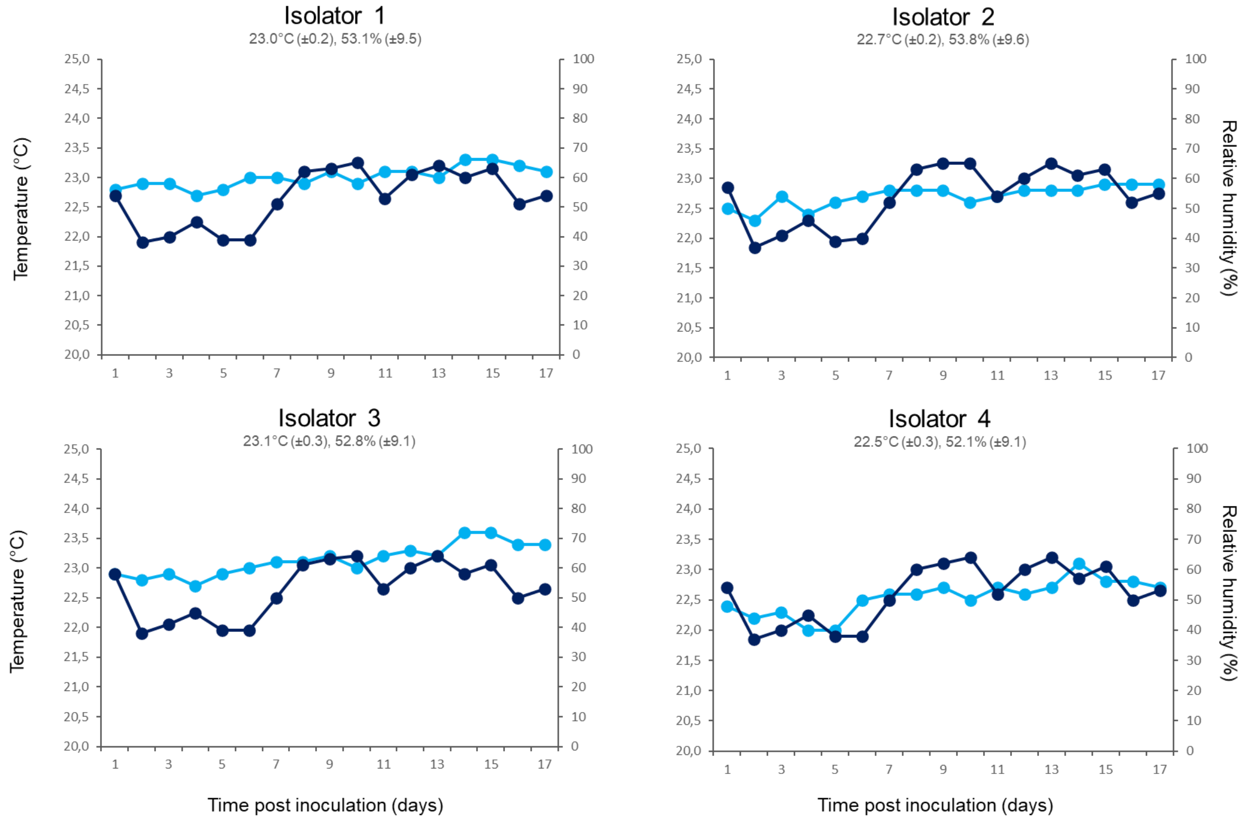
Isolator 1
23.0°C (±0.2), 53.1% (±9.5)
Isolator 2
22.7°C (±0.2), 53.8% (±9.6)
Isolator 3
23.1°C (±0.3), 52.8% (±9.1)
Isolator 4
22.5°C (±0.3), 52.1% (±9.1)
Temperature (°C)
Relative humidity (%)
Time post inoculation (days)

Figure S2. Environmental parameters in class III isolators. Graphs show temperature and relative humidity recordings in each isolator during SARS-CoV (a) and SARS-CoV-2 (b) transmission experiments. Light and dark blue circles and lines represent temperature and relative humidity measurements, respectively. Average values and standard deviation (±SD) are calculated from n=12 and n=17 recordings for SARS-CoV and SARS-CoV-2, respectively and shown below the title. Source data are provided as a Source Data file.
