# Supplementary material for: SARS-CoV and SARS-CoV-2 are transmitted through the air between ferrets over more than one meter distance
Source: Nat Commun. 2021 Mar 12;12:1653. doi: 10.1038/s41467-021-21918-6 (PMC7955093; doi:10.1038/s41467-021-21918-6)
Supplement: Supplementary file 3 — Reporting Summary [file 41467_2021_21918_MOESM3_ESM.pdf]

nature research

Corresponding author(s): Sander Herfst

Last updated by author(s): Feb 9, 2021

Reporting Summary

Nature Research wishes to improve the reproducibility of the work that we publish. This form provides structure for consistency and transparency in reporting. For further information on Nature Research policies, see Authors & Referees and the Editorial Policy Checklist .

Statistics

For all statistical analyses, confirm that the following items are present in the figure legend, table legend, main text, or Methods section.

n/a	Confirmed	
☐	☒	The exact sample size (*n*) for each experimental group/condition, given as a discrete number and unit of measurement
☐	☒	A statement on whether measurements were taken from distinct samples or whether the same sample was measured repeatedly
☒	☐	The statistical test(s) used AND whether they are one- or two-sided<br>*Only common tests should be described solely by name; describe more complex techniques in the Methods section.*
☒	☐	A description of all covariates tested
☒	☐	A description of any assumptions or corrections, such as tests of normality and adjustment for multiple comparisons
☐	☒	A full description of the statistical parameters including central tendency (e.g. means) or other basic estimates (e.g. regression coefficient) AND variation (e.g. standard deviation) or associated estimates of uncertainty (e.g. confidence intervals)
☒	☐	For null hypothesis testing, the test statistic (e.g. *F*, *t*, *r*) with confidence intervals, effect sizes, degrees of freedom and *P* value noted<br>*Give P values as exact values whenever suitable.*
☒	☐	For Bayesian analysis, information on the choice of priors and Markov chain Monte Carlo settings
☒	☐	For hierarchical and complex designs, identification of the appropriate level for tests and full reporting of outcomes
☒	☐	Estimates of effect sizes (e.g. Cohen's *d*, Pearson's *r*), indicating how they were calculated

Our web collection on statistics for biologists contains articles on many of the points above.

Software and code

Policy information about availability of computer code

Data collection	no custom software was used
Data analysis	CLC Genomics Workbench v20.0.3 (QIAGEN); Basic Variant Detection tool within the CLC Genomics Workbench.

For manuscripts utilizing custom algorithms or software that are central to the research but not yet described in published literature, software must be made available to editors/reviewers. We strongly encourage code deposition in a community repository (e.g. GitHub). See the Nature Research guidelines for submitting code & software for further information.

Data

Policy information about availability of data

All manuscripts must include a data availability statement. This statement should provide the following information, where applicable:

- Accession codes, unique identifiers, or web links for publicly available datasets
- A list of figures that have associated raw data
- A description of any restrictions on data availability

All data are available from the corresponding author (S.H.) on reasonable request. The source data underlying Figs. 2, 3, 4, 5, Figs. S1 and S2 are provided as a Source data file. The sequencing raw data were deposited in the NCBI Sequence Read Archive (SRA) under the BioProject PRJNA700531. Source data are provided with this paper.

Field-specific reporting

Please select the one below that is the best fit for your research. If you are not sure, read the appropriate sections before making your selection.

Life sciences study design

All studies must disclose on these points even when the disclosure is negative.

Sample size	For ethical considerations, the group size of animal experiments is small. A group of 8 ferrets was used per virus: four donor ferrets and four indirect recipient ferrets. The results of the transmissions studies are qualitative. Therefore, no sample size calculation was performed.
Data exclusions	No data were excluded from the analyses
Replication	For the transmission studies, data were replicated by having four independent transmission experiments per virus (see sample size). All attempts at replication were successful with respect to the virus replication in the donor ferrets, but variation in transmission efficiency was observed as expected.
Randomization	Ferrets were randomly allocated to each group.
Blinding	Investigators were blinded while performing RNA isolation, qRT-PCR, virus titration, deep sequencing and ELISA. For transmission experiments blinding was not possible due to biosafety reasons.

Reporting for specific materials, systems and methods

We require information from authors about some types of materials, experimental systems and methods used in many studies. Here, indicate whether each material, system or method listed is relevant to your study. If you are not sure if a list item applies to your research, read the appropriate section before selecting a response.

Materials & experimental systems

n/a	Involved in the study	
☐	☒	Antibodies
☐	☒	Eukaryotic cell lines
☒	☐	Palaeontology
☐	☒	Animals and other organisms
☒	☐	Human research participants
☒	☐	Clinical data

Methods

n/a	Involved in the study	
☒	☐	ChIP-seq
☒	☐	Flow cytometry
☒	☐	MRI-based neuroimaging

Antibodies

Antibodies used	goat anti-ferret IgG HRP labelled, ab112770, Abcam
Validation	The antibody was used according to the manufacturer instructions.

Eukaryotic cell lines

Policy information about cell lines

Cell line source(s)	Madin-Darby Canine Kidney (MDCK) cells (ATCC), Vero-E6 cells (ATCC)
Authentication	None of the cell lines were authenticated
Mycoplasma contamination	All cell lines tested negative for mycoplasma
Commonly misidentified lines (See ICLAC register)	None of the cell lines used in this study have been identified as commonly misidentified lines

Animals and other organisms

Policy information about studies involving animals; ARRIVE guidelines recommended for reporting animal research

Laboratory animals	Mustela putorius furo, female, 6-month old
Wild animals	The study did not involve wild animals
Field-collected samples	The study did not involve samples collected from the field
Ethics oversight	Research was conducted under a project license from the Dutch competent authority (Centrale Commissie Dierproeven (CCD), license number AVD1010020174312) and the study protocols were approved by the institutional Animal Welfare Body (Erasmus

MC permit number 17-4312-03, 17-4312-05 and 17-4312-06).

Note that full information on the approval of the study protocol must also be provided in the manuscript.
